# The Sign 4 Big Feelings Intervention to Improve Early Years Outcomes in Preschool Children: Outcome Evaluation

**DOI:** 10.2196/25086

**Published:** 2022-05-20

**Authors:** Rosemary Davidson, Gurch Randhawa

**Affiliations:** 1 Institute for Health Research University of Bedfordshire Luton United Kingdom

**Keywords:** language development, sign language, early years outcomes, well-being

## Abstract

**Background:**

Any delays in language development may affect learning, profoundly influencing personal, social, and professional trajectories. The effectiveness of the Sign 4 Big Feelings (S4BF) intervention was investigated by measuring changes in early years outcomes (EYOs) after a 3-month period.

**Objective:**

This study aims to determine whether children’s well-being and EYOs significantly improve (beyond typical, expected development) after the S4BF intervention period and whether there are differences between boys and girls in progress achieved.

**Methods:**

An evaluation of the S4BF intervention was conducted with 111 preschool-age children in early years settings in Luton, United Kingdom. Listening, speaking, understanding, and managing feelings and behavior, in addition to the Leuven well-being scale, were assessed in a quasi-experimental study design to measure pre- and postintervention outcomes.

**Results:**

Statistically and clinically significant differences were found for each of the 7 pre- and postmeasures evaluated: words understood and spoken, well-being scores, and the 4 EYO domains. Gender differences were negligible in all analyses.

**Conclusions:**

Children of all abilities may benefit considerably from S4BF, but a language-based intervention of this nature may be transformational for children who are behind developmentally, with English as an additional language, or of lower socioeconomic status.

**Trial Registration:**

ISRCTN Registry ISRCTN42025531; https://doi.org/10.1186/ISRCTN42025531

## Introduction

### Background

Any delays in language development may affect speech and learning, profoundly influencing personal, social, and professional trajectories. The role of social interaction and gesturing in cognitive development is paramount [1]. Socioemotional development is increasingly acknowledged as important for future life opportunities. *Effective mastery* of social and emotional skills supports the attainment of key life outcomes such as good health and social well-being, educational attainment and employment, and the avoidance of behavioral and social difficulties [2], especially in the context of increasing concern about children’s mental health and well-being [3,4]. Gesturing has been proposed as a *therapeutic communication tool* to help children express emotions and construct an understanding of their own internal states [5].

Goodman et al [6] linked social, emotional, and cognitive skills recorded in the British Cohort Study from children born in 1970 aged 10 years with their experiences 32 years later. Developing a good range of cognitive, social, and emotional skills—including good emotional well-being, self-regulation, and a sense of self-efficacy—in childhood is important for success in adult life. Moreover, psychological problems experienced in childhood affect the ability to work in adulthood and impair earning power, marital stability, and intergenerational and within-generation social mobility [7]. In terms of ensuring school readiness, it is argued that developing preschool children’s socioemotional skills as well as language skills help them adjust to primary school. This is argued as particularly important for at-risk children as a way of ensuring school readiness [8].

### Evidence for Effectiveness of Sign Language and Gesturing in Children

Research on the benefits of sign language for hearing children spans a range of ages. Góngora and Farkas [9] reported how an infant sign-language program with babies aged between 5 and 9 months increased visual and tactile interactions and the likelihood of vocal interactions compared with mother-infant dyad control groups. However, this was a small sample of 14 children with mothers from middle to higher socioeconomic status. Children as young as 12 months were reported to be able to comprehend communicative intentions behind gestures [10], and Vigliocco et al [11] found that preverbal children who have an understanding of both gesture and word combinations then go on to acquire the equivalent word combinations. In other words, gestures come before verbal speech. This is also argued in research investigating the relationship between motor and language development, in that infants practice with motor skills first—gestures—as a precursor to using new vocabulary [12]. In the late 1990s, Felzer [13] reported that hearing preschool children retained far more words and phonetic sounds using a multisensory approach by learning to read by seeing, hearing, saying, and signing words. Gesturing in particular was tested by Cook and Goldin-Meadow [14] during teaching, which in turn encouraged children to mirror gestures, increasing engagement and interaction, thereby enhancing learning.

School-based research has shown that teachers who use sign language with young hearing children significantly increased their vocabularies compared with children taught conventionally. These positive effects were sustained through the following kindergarten year [15]. A later study by the same researcher used American Sign Language with hearing children and reported that they had made significant progress in vocabulary [16]. In terms of specific academic subjects, children who observed gestures while learning mathematics performed better than a matched control group who received verbal instruction only [17]. In terms of longitudinal evidence, Rowe and Goldin-Meadow [18] reported that the gestures children make at 18 months can predict later language learning. They found that gestures used at 18 months predicted vocabulary at 42 months, and gestures and speech combinations conveying sentence-like constructs at 18 months predicted sentence complexity at 42 months.

With respect to the neuroscientific evidence, brain scanning studies have shown that the same areas of the brain are activated for symbolic gestures, signs, and words, and gestures conveying meaning will activate these parts of the brain, thus making learning new words easier [19,20]. The sensorimotor stage—birth to 2 years—extends from birth to early language development [21]. Children gradually construct knowledge by coordinating their vision and hearing with physical interactions. In the preoperational stage (2-7 years), children can think symbolically, and a gesture can stand for something other than simply moving hands. As the understanding of brain function has advanced through theories such as embodied cognition [22], cognitive processes have been linked to our physical interactions with the world and the idea that signing and gestures may facilitate learning.

Although there is a great deal of research on the positive effects of signing and gesturing, there is also evidence to the contrary. A randomized controlled trial on the effects of signing on infant language reported no acceleration in linguistic development; however, mothers were more responsive to their child’s nonverbal cues [23]. Concerns have also been raised about whether gesturing actually hampers language development in preverbal children. However, Goodwyn et al [24] reported that symbolic gesturing does not hamper verbal development and may encourage it.

### Sign 4 Program, Luton, United Kingdom

Luton is a large town situated in the county of Bedfordshire and 50 km northwest of London. It is 1 of 3 White British minority towns in the United Kingdom, extremely ethnically diverse because of high rates of international immigration and a greater number of people moving from London boroughs to Luton because of changes in benefit entitlements. Nearly one-third of children in Luton live in poverty (28.5%) [25]. More than a quarter of children are classified as obese (25.9%), and levels of General Certificate of Secondary Education (GCSE) attainment are worse than average for England overall. The dental decay rates in 5-year-olds in Luton (36.8%) are significantly higher than the England average [26]. Luton has a sizable transient, vulnerable population, with many families living in temporary accommodation. It is estimated that between 30% and 50% of the population were either not born or not living in Luton at the time of the 2011 census [27].

Consequently, multiple languages are spoken, and the number of primary school pupils with English as an additional language (EAL) now outnumbers English-speaking pupils [28]. To meet these challenges, Luton Borough Council (LBC) formulated an early help strategy to support stakeholders in a coordinated way [28]. LBC funds Flying Start, an organization dedicated to improving early years outcomes (EYOs) through support, programs, and services [29]. Sign 4 Big Feelings (S4BF) is one of many interventions funded by Flying Start [30].

This study investigates the effectiveness of the S4BF intervention following initial pilot data reporting accelerated progress in preschool children. Four interventions have been identified for evaluation, of which S4BF is one [31-36]. We examine its impact with an analysis of pre- and postoutcome data collected from Luton settings. The aims of this study are as follows:

- Do EYOs significantly improve (beyond typical, expected development) after using the S4BF program?
- Is there a statistically significant relationship between EYO domains (listening and attention, feelings and behavior, speaking, and understanding), EYO domains and children’s age, and the Leuven well-being scale?
- Does children’s well-being improve after the S4BF intervention period?
- To what extent does gender play a role in any progress made?

## Methods

### Ethics Approval and Consent to Participate

This study was approved by the University of Bedfordshire research ethics committee (UREC104) on April 10, 2017. Written consent was obtained from the parents.

### Availability of Data and Materials

The data sets used and analyzed during this study are available from the corresponding author upon reasonable request.

### Participants

Data from 111 children were collected. Boys outnumbered girls (60/111, 54% and 48/111, 43.2%, respectively; 3/111, 3% unknown), just over a third of children had English as a second language (37/111. 33.3%), and just over one-fifth had funded preschool places (25/111, 22.5%; Table 1). At the start of the study, the mean age of the children was 39 (SD 10.81) months, but the ages ranged from 21 to 71 months. A total of 110 wordlists (words understood and spoken pre- and postintervention), 91 EYO assessments (pre- and postintervention), and 48 Leuven well-being scales (pre- and postintervention) were completed.

**Table 1:** Children by age, gender, English as an additional language, and funded 2 status (N=111).

Characteristics		Values
**Gender, n (%)**		
	Male	60 (54.1)
	Female	48 (43.2)
	Unknown	3 (2.7)
**EAL^a^ status, n (%)**		
	EAL-yes	37 (33.3)
	EAL-no	45 (40.5)
	Unknown	28 (25.2)
**Funding status, n (%)**		
	Funded 2 yes	25 (22.5)
	Funded 2 no	82 (73.9)
	Unknown	3 (3.6)
**Age (months), mean (SD; range)**		
	January	39.44 (10.81; 21-71)
	March	42.65 (11.11; 44-75)

^a^EAL: English as an additional language.

The S4BF intervention was delivered by childminders or early years practitioners trained as designated safeguarding officers (DSOs) overseeing preschool-age children in early years settings across Luton. Registered childminders provide childcare to young children in their own homes and must meet a range of statutory requirements set out by the Office for Standards in Education, Children’s Services and Skills and LBC, such as safety standards and rules regarding care. DSOs are early years practitioners working in private, voluntary, or independent nursery settings. Their role is to keep abreast of relevant legislation, remain up-to-date with training related to safeguarding issues, and consequently be able to identify any sign of abuse, maltreatment, neglect, or distress in preschool children.

### Recruitment

Childminders and DSOs attend termly meetings held by LBC for briefings and changes in legislation and training. A slot was organized for the head of S4BF and the sign language trainer to give a presentation on S4BF, followed by a demonstration, hand out of S4BF books and dolls, and time for childminders and DSOs to practice the stories and sign among themselves. If childminders and DSOs were responsible for children aged 2 to 5 years and happy to participate, a memorandum of understanding was given with instructions on how to complete forms to monitor progress before and 3 months after S4BF, when outcome forms would be returned for analysis. Forms required childminders and DSOs to record EYOs in listening and attention, understanding, speaking, and managing feelings and behavior.

### Data Collection

Childminders and DSOs were asked to choose 2 children to monitor for the study period and collect and submit data at the beginning and end of the term. Where possible, these children were to have lower levels of expected development in communication and language and concerns about well-being as they were viewed as able to benefit most from the S4BF intervention. Part of the data collection was a statutory requirement, that is, the submission of EYO scores to monitor development and 2 additional forms per child completed as part of the evaluation: the number of words understood and spoken and the Leuven well-being scale. As DSOs work in nursery settings, they have a number of children of varying abilities and circumstances to monitor. However, childminders have a more limited scope as they usually care for between 1 and 3 children. Childminders and DSOs were briefed on how to complete and submit the forms as part of the termly meetings (see the *Recruitment* section above). As forms were submitted to the research project, they were checked to ensure that any queries could be addressed by settings and resolved as quickly as possible.

### Outcome Measures

Practitioners were already familiar with EYO scales to monitor early years’ progress as they typically record this information every term. Children are assigned to age bands (ie, 24-30 months and 32-40 months), which are then subdivided into c=low, b=secure, and a=high to rate the level of attainment within each age band. Practitioners rate children on their ability; therefore, a child’s chronological age may not reflect where they are placed on the EYO scale. Children are expected to move up 1 *level* per term; for example, 16-22c (low) to 16-22b (secure), which represents expected progress.

Two further measures were collected: the number of words (1) understood and (2) spoken out of 16 keywords featured in the S4BF storybook (happy, sad, why, because, quiet, hiding, crying, excited, frightened, dangerous, safe, worried, secret, shouting, noisy, and proud) and the Leuven well-being scale (for childminders and DSOs to fill in if they were trained to do so). A total of 2 Leuven scales exist for well-being and involvement, respectively. For the purpose of this study, the Leuven well-being scale was used [37,38] because it is commonly used by early years professionals in Luton. The Leuven scales were developed in part because it was hypothesized that where there are consistently low levels of well-being and involvement, it is likely that a child’s development will be compromised [39,40]. The Leuven scale allows early years practitioners to place each child on a 5-point well-being scale ranging from 1 (extremely low) to 5 (extremely high), with clear definitions at each point for practitioners to judge against; training in the use of the scales is also routinely provided for Luton’s early years’ workforce.

Further information was collected on children’s gender, age in months, whether EAL, and whether they have funded early years status (funded 2), which was used as a marker of deprivation.

### Research Design

A quasi-experimental design was used in this study. Data were collected pre- and postintervention from childminders and DSOs in Luton. Where possible, the children assessed were those with lower levels of expected development in communication and language and those subject to concerns about well-being because they would benefit the most from the S4BF intervention.

### Statistical Tests

A paired sample 2-tailed *t* test was used to measure the progress made by children, comparing EYO scores collected at the beginning and end of the school term, to determine whether the progress was significant. A correlation analysis was performed to determine any positive and statistically significant relationships between the variables under study. An analysis of covariance (ANCOVA) was conducted on the mean EYO scores to determine the level of progress achieved by preschool children when controlling for age.

### Sign 4 Big Feelings Intervention

The S4BF intervention was developed to address gaps of attainment in preschool children. Such gaps were identified by looking at routinely collected early years data to monitor the progress of children across Luton. S4BF uses books depicting children experiencing different emotions and accompanying dolls to *act out* how the characters feel. Early years practitioners read books to children regularly at storytimes using simple sign language to convey the emotions of the characters in the story and encourage children to copy the signs and repeat the words the signs convey (“Ishan feels really safe. Why does he feel safe? Because he’s having a bedtime story;” “Izzy is sad. Why is she sad? Because the television is broken”).

The intervention was designed to help preschool children to communicate more effectively, express emotions constructively, and learn linking words such as *because* and *why* to use complex sentences to explain the reasons behind behaviors. S4BF was designed to provide young children with a range of vocabulary to convey how they feel and an opportunity to talk with a trusted adult about events they may find frightening or difficult, such as family conflict or domestic abuse. The intervention was intended to help children 3-fold: improve speech and vocabulary with stories and accompanying sign language, help them *name and tame* their emotions rather than act out with difficult or destructive behavior, and help early years practitioners in identifying any safeguarding issues that may arise by talking about the emotions depicted in the S4BF book. Textbox 1 shows the theory of change to illustrate the progression from S4BF outputs and activities to short- and long-term outcomes.

**Theory of change: use of a sign-language intervention to improve early years outcomes in preschool children.**

Outputs

- Funding (by local government) and development of sign-language program to improve communication via speech and language development through:
  Sign language to reinforce keywords with accompanying gestures and facial expressions
  Stories to reflect different social situations
  Repetition of stories and use of signs during interactions during the school day
  Production of Sign 4 Big Feelings books and dolls

Activities

- Training sessions with early years practitioners in the Sign 4 Big Feelings program
- Early years practitioners read Sign 4 Big Feelings books and used dolls with children at regular story times throughout the week
- Recording and monitoring of early years outcomes over time to ensure disadvantaged children keep up with national average development and attainment goals

Short-term outcomes

- Early years practitioners are trained in key sign-language skills
- Accelerated improvements in key early years outcomes:
  Listening and attention
  Understanding
  Speaking
  Managing feelings and behavior
- Significant improvements in:
  Number of words spoken and understood
  Well-being
- Narrowing the gap in attainment with peers before starting primary school for those children who are developmentally behind

Longer-term outcomes

- Early years practitioners can use their sign-language skills with future cohorts of children
- Early years settings appreciate long-term benefits of the use of sign-language intervention to improve early years outcomes and embed as part of long-term provision
- Improved educational attainment, social skills, and employment prospects, leading to a better quality of adult life and better health

## Results

### Overview

Table 2 summarizes the S4BF data set. Although 111 children took part in the evaluation of S4BFs, some measures were incomplete.

In total, 48 Leuven Scales (pre- and postintervention), 91 EYO assessments (pre- and postintervention), and 110 word lists (words understood and spoken pre- and postintervention) were completed. Baseline data were collected in November 2016, and follow-up data were collected 3 months later, from February to March 2017.

**Table 2:** Summary of the Sign 4 Big Feelings data set (N=111).

Outcome			Valid, n (%)		Missing, n (%)		Mean (SD)		Range		Minimum		Maximum
**January**													
	Words understood	110 (99.1)		1 (0.9)		6.30 (4.04)		16		0		16	
	Words said	110 (99.1)		1 (0.9)		4.10 (3.93)		16		0		16	
	Leuven	48 (43)		63 (57)		2.89 (1.05)		4		1		5	
	EYO^a^ listening and attention	91 (82)		20 (18)		9.06 (2.45)		11		4		15	
	EYO understanding	91 (82)		20 (18)		8.83 (2.62)		13		2		15	
	EYO speaking	91 (82)		20 (18)		7.94 (2.96)		14		1		15	
	EYO feelings and behavior	91 (82)		20 (18)		8.71 (2.31)		10		4		14	
**March**													
	Words understood	110 (99.1)		1 (0.9)		11.41 (3.57)		13		3		16	
	Words said	110 (99.1)		1 (0.9)		9.60 (4.44)		16		0		16	
	Leuven	48 (43)		63 (57)		3.97 (0.73)		3		2		5	
	EYO listening and attention	91 (82)		20 (18)		11.02 (2.44)		12		6		18	
	EYO understanding	91 (82)		20 (18)		10.77 (2.45)		13		5		18	
	EYO speaking	91 (82)		20 (18)		10.25 (2.69)		14		4		18	
	EYO feelings and behavior	91 (82)		20 (18)		10.69 (2.27)		11		6		17	

^a^EYO: early years outcome.

### Analysis

In addition to descriptive statistics, a number of tests were used: a within samples *t* test, correlations, ANCOVAs, and multivariate analysis of covariance.

#### Paired Sample t Test

A paired sample *t* test was conducted to establish any statistically significant difference between the pre- and post-S4BF intervention after checking that the data were within the normal distribution (Table 3). There was a statistically significant difference in the mean scores for each of the 7 pre- and postpairs tested. For mean words understood by children, a paired-samples *t* test indicated that scores were significantly higher in March (mean 11.41, SD 3.57) than in January (mean 6.3, SD 4.04; *t*_109_=16.4; *P*<.001; Cohen *d*=1.56). Mean words spoken were significantly higher in March (mean 9.6, SD 4.44) than in January (mean 4.11, SD 3.93; *t*_109_=15.55; *P*<.001; Cohen *d*=1.38). The Leuven well-being scores were significantly higher in March (mean 3.98, SD 0.73) than in January (mean 2.89, SD 1.05; *t*_47_=9.78; *P*<.001; Cohen *d*=1.42). The EYO listening and attention was significantly higher in March (mean 11.0, SD 2.45) than in January (mean 9.06, SD 2.45; *t*_90_=12.46; *P*<.001; Cohen *d*=1.3).

The EYO understanding was significantly higher in March (mean 10.75, SD 2.45) than in January (mean 8.83, SD 2.62; *t*_90_=11.64; *P*<.001; Cohen *d*=1.2). The EYO speaking was significantly higher in March (mean 10.25, SD 2.69) than in January (mean 7.94, SD 2.96; *t*_90_=11.27; *P*<.001; Cohen *d*=1.17). The EYO feelings and behavior were significantly higher in March (mean 10.69, SD 2.27) than in January (mean 8.71, SD 2.31; *t*_90_=11.9; *P*<.001; Cohen *d*=1.24).

**Table 3:** Paired-samples *t* test results.

Pairs	Paired differences				*t* test (*df*)		*P* values (2-tailed)
	Mean (SD)	SE mean	95% CI of the difference				
1. Words Jan under–words Mar under	−5.11 (3.26)	0.31	−5.73 to −4.50	−16.43 (109)		<.001	
2. Words Jan say–words Mar say	−5.49 (3.70)	0.35	−6.19 to −4.79	−15.55 (109)		<.001	
3. Leuven Jan–Leuven Mar	−1.08 (0.76)	0.11	−1.30 to −0.86	−9.78 (47)		<.001	
4. EYO^a^ Jan list and att–EYO Mar list and att	−1.95 (1.49)	0.15	−2.26 to −1.64	−12.46 (90)		<.001	
5. EYO Jan under–EYO Mar under	−1.92 (1.60)	0.16	−2.25 to −1.59	−11.46 (90)		<.001	
6. EYO Jan speak–EYO Mar speak	−2.30 (1.95)	0.20	−2.71 to −1.90	−11.27 (90)		<.001	
7. EYO Jan feel and beh–EYO Mar feel and beh	−1.97 (1.58)	0.16	−2.30 to −1.64	−11.91 (90)		<.001	

^a^EYO: early years outcome.

#### Correlations

A correlation analysis was undertaken for children’s age, EYOs, and Leuven well-being scales (Table 4). There was a positive, statistically significant relationship between age and the EYO of listening and attention Pearson *r*_91_=0.56, *P*<.001; age and EYO for understanding, Pearson *r*_91_=0.57, *P*<.001; age and EYO for speaking, Pearson *r*_91_=0.49, *P*<.001; and age and EYO for feelings and behavior, Pearson *r*_91_=0.51, *P*<.001. A strong positive, statistically significant relationship was also found between each of the EYOs; for example, EYOs for listening and attention and understanding, Pearson *r*_91_=0.90, *P*<.001, and between feelings and behavior and speaking, Pearson *r*_91_=0.83, *P*<.001. A positive, statistically significant relationship was found between the Leuven well-being scores and all the EYO domains, the strongest of which was with speaking, Pearson *r*_44_=0.51, *P*<.001.

**Table 4:** Correlations between age, early years outcomes, and Leuven scales.

			Age March		EYO^a^ March listening and attention		EYO March understanding		EYO March speaking		EYO March feelings and behavior
**Age March**											
	Pearson correlation	1		0.56^b^		0.57^b^		0.49^b^		0.51^b^	
	Significance (2-tailed)	N/A^c^		0.00		0.00		0.00		0.00	
	n	110		91		91		91		91	
**EYO March listening and attention**											
	Pearson correlation	N/A		N/A		0.90^b^		0.87^b^		0.87^b^	
	Significance (2-tailed)	N/A		N/A		0.00		0.00		0.00	
	n	N/A		N/A		91		91		91	
**EYO March understanding**											
	Pearson correlation	N/A		N/A		N/A		0.85^b^		0.82^b^	
	Significance (2-tailed)	N/A		N/A		N/A		0.00		0.00	
	n	N/A		N/A		N/A		91		91	
**EYO March speaking**											
	Pearson correlation	N/A		N/A		N/A		N/A		0.83^b^	
	Significance (2-tailed)	N/A		N/A		N/A		N/A		0.00	
	n	N/A		N/A		N/A		N/A		91	
**Leuven March**											
	Pearson correlation	N/A		0.48^b^		0.52^b^		0.58^b^		0.50^b^	
	Significance (2-tailed)	N/A		0.00		0.00		0.00		0.00	
	n	N/A		44		N/A		N/A		N/A	

^a^EYO: early years outcome.

^b^Correlation significant at the 0.01 level (2-tailed).

^c^N/A: not applicable.

#### EYO Progress, Gender, English as an Additional Language, Funded 2 Status, and Well-being

Although there was no difference in gender in terms of progress, children with EAL accomplished nearly 4 steps in EYO stages (boys 3.7 steps, girls 3.8), whereas non-EAL children accomplished 2 steps (both boys and girls progressed 2.2 steps). Expected progress per term (3 months) is one step.

Gender differences were marginal, with funded 2 girls making slightly more progress than boys (girls 4.4 steps and boys 3.9) and nonfunded boys making slightly more progress than nonfunded girls (boys 2.6 steps, girls 2.4 steps). Overall, children who had funded 2 status progressed just over 4 steps (4.15) compared with nonfunded children who progressed 2.5 steps. In terms of well-being, children were assessed on the Leuven scale pre- and postintervention in January and March. At baseline, most children were put at level 3 (moderate, 46%), and at level 4 (high, 54%) postintervention, showing an overall shift of the sample higher up the Leuven well-being scale.

#### Analysis of Variance

An analysis of variance was conducted on the S4BF data set (Table 5). A 3×2 within-group ANCOVA was run on the means of the EYO scores as the scores correlated with one another. The ANCOVA conducted with mean EYO scores (mean 10.73, SD 2.33) showed that the main effect of EYOs was statistically significant throughout time when controlling for age (*F*_1,89_=4.89, *P*=.03; partial η 0.58; 39.33, SD 10.81). There was a statistically significant interaction between EYOs (mean 10.73, SD 2.33) and age (*F*_1,89_=6.18, *P*=.01; partial η 0.72; mean 39.33, SD 10.81). There was a statistically significant interaction between EYOs (mean 10.73, SD 2.33) and EAL (*F*_1,89_=8.48, *P*=.005; partial η 0.09; mean 4.33, SD 2.29). There was another statistically significant interaction in the ANCOVA test between EYOs (mean 10.73, SD 2.33) and funded 2 status (*F*_1,89_=10.65, *P*=.002; partial η 0.11; mean 10.84, SD 2.29). No statistically significant interaction was found between EYOs and gender or between combinations of the aforementioned variables.

**Table 5:** Analysis of covariance with mean EYO^a^ scores.

Tests of within-subjects effects	Type III sum of squares	Mean square	*F* (df)	Significance	Partial η squared
EYO	4.011	4.011	4.895 (1)	0.030	0.058
EYO×age_Jan	5.066	5.066	6.183 (1)	0.015	0.072
EYO×gender	0.011	0.011	0.014 (1)	0.907	0.000
EYO×EAL^b^	6.955	6.955	8.488 (1)	0.005	0.096
EYO×funded 2	8.726	8.726	10.651 (1)	0.002	0.117
EYO×gender×EAL	0.008	0.008	0.010 (1)	0.922	0.000
EYO×gender×funded 2	0.209	0.209	0.255 (1)	0.615	0.003
EYO×EAL×funded 2	0.000	0.000	0.000 (1)	0.997	0.000
EYO×gender×EAL×funded 2	0.016	0.016	0.019 (1)	0.891	0.000

^a^EYO: early years outcome.

^b^EAL: English as an additional language.

### Statistical Significance and Clinical Significance: EYO Scores

The previous section has shown that children made statistically significant progress in their EYO scores. Children are expected to progress by 1 EYO *level* per term, but to what extent have children progressed further than this? Further inspection of the data showed that children cared for by childminders showed considerably less progress (1.4 steps) compared with the EYO results reported by DSOs (3.6 steps).

### Control Data

Control data collected from the same academic year records average progress of preschool children throughout 2 terms. Table 6 shows that children made 1 or 2 steps progress in each EYO domain. Children are expected to progress 1 step in each term. Therefore, data collected from these settings show that below-expected progress was made in listening and attention and speaking (1 step throughout 2 terms), and expected progress was made in understanding and managing feelings and behavior (2 steps throughout 2 terms).

**Table 6:** Control data: average steps progress of children throughout 2 school terms.

EYO^a^ domain	Autumn term 1, n (%)					Spring term 2, n (%)				
	Total pupils	Below	At	Above	Total pupils		Below	At	Above	Steps progress
Listening and attention	413 (100)	140 (33.9)	190 (46)	82 (19.9)	498 (100)		130 (26.1)	189 (38)	179 (35.9)	1
Understanding	413 (100)	169 (40.9)	169 (40.9)	75 (18)	498 (100)		179 (35.9)	169 (33.9)	150 (30.1)	2
Speaking	404 (100)	186 (46)	162 (40)	56 (13.9)	498 (100)		220 (44.2)	150 (30.1)	130 (26.1)	1
Managing feelings and behavior	394 (100)	158 (40.1)	189 (47.9)	47 (11.9)	498 (100)		189 (38)	189 (38)	120 (24.1)	2

^a^EYO: early years outcome.

## Discussion

### Principal Findings

Statistically significant differences were found for each of the seven pre- and postmeasures taken: words understood and spoken, well-being scores, and the 4 EYO domains. Therefore, children achieved better than expected progress when assessed at the end of the intervention. Most children in the sample achieved better than expected progress, with many progressing multiple steps in EYO attainment.

Positive correlations were found between age and the EYOs of listening and attention, understanding, speaking, and feelings and behavior. A strong, statistically significant relationship was found between each EYO domain. Therefore, having a high EYO in one domain was positively associated with having a high score in another. This is corroborated by early years professionals, in that one EYO domain underpins another. For example, if a child is assessed as low in understanding or speaking, they are unlikely to manage their feelings and behavior as that involves speaking. A positive, statistically significant relationship was found between the Leuven well-being scores and all the EYO domains, the strongest of which was with speaking. In other words, there was a positive relationship between well-being and higher EYO scores. Children with EAL made more progress than native speakers, and those with funded places made more progress than nonfunded children.

As age was positively correlated with all EYO domains, an ANCOVA was run with age as a covariate. This showed that EYO as a main effect was statistically significant throughout time when controlling for age. There was a statistically significant interaction between EYO and EAL and between EYO and funded 2 status. No statistically significant interaction was found between EYO and gender. Gender differences were negligible in all analyses undertaken. Considerable variations in progress were evident when comparing data reported by childminders and DSOs.

Clinically significant results in EYO scores were shown by documenting the stages of progress made by children in the sample. Whereas more than one-quarter of children made expected progress (by progressing up 1 level), most progressed more than expected (by 2 stages or more). Although children reported as having progressed by multiple stages should be treated with caution, overall, most children in the sample made better than expected progress in terms of these 4 EYO domains. Indeed, in the early years, professionals reported seeing rapid progress in children if they were engaged in learning.

In summary, the statistical tests show us that the children made significant progress in terms of EYOs and words understood and spoken, even when controlling for the effect of age. By looking at the number of stages of progress made, we know that a large proportion of the sample made clinically significant progress.

### Limitations

There was a clear divide in the level of progress reported by childminders and DSOs. Childminders are likely to care for children on a part-time basis, which means less contact and therefore less time to read stories and sign consistently with children. Childminders seem less supported in that they work from home compared with DSOs who work within an institution and consequently have support and input from colleagues. They also care for smaller groups of children, some of whom may not have met the study criteria of being below the level of expected development. A number of childminder forms were excluded from the sample as insufficient detail was provided on the EYOs (eg, the age range in months was given, but not an a-c rating to indicate proficiency within that band or only an a-c rating without an age band given in months). Childminders may be less supported as independent businesses than as part of institutions, and although they were provided training and guidance on how to use the EYO bands, a proportion were still unable or unsure how to report them fully. Conversely, there may have been a positive bias in the results reported by DSOs, as the progress made by some children seemed so great, moving many EYO bands further ahead.

A relatively small sample size was achieved; however, we were interested in studying a subsection of that population, specifically children below the expected levels of development. On the basis of the local authority tracking system for EYOs, a step up the scale per term is judged as typical progress; however, there is no such thing as the *typical* child. In fact, funded 2 children are expected to accelerate progress to catch up. Early years settings in Luton report that they are getting better at identifying children who require additional help and are modifying their approach to better meet educational needs with interventions such as S4BF. For those children who were reported to have made less than expected progress, there may be an undiagnosed special educational need, inconsistent attendance (2-year-olds are nonstatutory), or change in key workers as possible explanations. Data on well-being as measured on the Leuven scale were particularly restricted, as not all early years practitioners were trained to use them. Scales are an indicator of progress, but they do not consider each child as a whole and their personal circumstances.

Childminders and DSOs were asked to incorporate the S4BF book and doll into their daily routines; however, there were variations between practitioners and settings regarding the frequency of use. In particular, childminders commented that the intervention period was too short to allow for any substantive change in children. Future studies would benefit from running across more than 1 term and having larger sample sizes.

An independent assessment of child outcomes would have strengthened the results. However, staff in early years settings routinely assess children in terms of EYO progress as an integral part of their role. Furthermore, it could be argued that staff working with children on a daily basis would be best placed to judge the rate, extent, and nuances of children's progress than an external assessor.

This study has been defined by the progress made by children rather than final attainment. Further work is required to ascertain whether the considerable progress that most children in this sample have made has allowed them to catch up with their peers, although this approach would benefit from a study tracking children for longer than 3 months. A longer-term, longitudinal study tracking children’s educational outcomes up to secondary school may help understand critical points in development and where gender differences start to be apparent. It is hoped that the positive effects reported here will be sustained throughout time, as with Daniels [15], and have a profound and long-lasting impact [18].

### Previous Research and Theory

Overall, the results reported here contribute to research on the positive effects of sign language and gesturing for preschool children [11,12]. Effects of low income on educational outcomes [41,42] have been reported. The results presented here suggest that children receiving funding (funded 2) made considerable progress. At the very least, interventions such as S4BF encourage children and early years practitioners to engage with each other through gestures, eye contact, and facial expressions, fostering receptive, word-rich environments [43]. It could be argued that the benefits of interventions successful in promoting speech, sentences, and acquisition of vocabulary are difficult to quantify in the sense that children are given the tools to quickly build upon iteratively, influencing more than educational attainment. The UK Government has expanded funded preschool places in recent years, and it is an area where inequalities in longer-term outcomes can be tackled early on [44].

Children learning a foreign language also face greater challenges in attainment than their peers [45,46]. The results presented here from the S4BF intervention suggest that EAL children can make significant progress beyond expected levels of attainment, thereby helping them catch up with their peers. Gender did not appear to have any significant effect on EYOs, wordlists, or well-being. Any differences between boys and girls are perhaps less pronounced in the early years in terms of educational outcomes; however, we know that they become stark by the time children reach early adulthood [47]. The statutory data collected in Luton suggest that the gap becomes apparent at reception age when children are required to write and therefore have fine motor skills.

Working with Flying Start, LBC, and early years practitioners to evaluate S4BF has facilitated the cooperation of people working in settings with the expertise to work closely with young children. Progress has been measured in multiple domains: EYOs, word knowledge, and Leuven well-being scales. EYOs, in particular, are established measures in early years education and widely used across Luton, allowing professionals to place children on a reasonably finely grained scale to monitor their progress on a range of key developmental domains.

### Conclusions

S4BF was designed in part to help children express difficult emotions, thereby reducing destructive behavior. Most children in this sample made significant progress in all EYO domains, not least managing feelings and behavior. This element needs to be explored further in the forthcoming process study of S4BF. The wider evaluation of the Sign 4 Program will include the views of parents [48] and in-depth interviews with early years professionals. It will also be possible to see if there has been an increase in safeguarding referrals since the introduction of S4BF. The evidence presented here suggests that interventions such as S4BF can benefit preschool children but are particularly important for children who experience multiple disadvantages. S4BF was developed because of the worrying gaps in the attainment of young children in Luton. Such children are starting at a disadvantage and appear to benefit greatly from this additional support. Children of all abilities may benefit considerably from S4BF. However, the intervention may be transformational for children who are behind developmentally, with EAL needs or of lower socioeconomic status, and who require additional support to catch up with their peers to realize their potential in later life.

## Abbreviations

DSO: designated safeguarding officer
EAL: English as an additional language
EYO: early years outcomes
LBC: Luton Borough Council
S4BF: Sign 4 Big Feelings
